# In vitro testing of titanium knot fastener used in cardiac and general surgery with pull apart force

**DOI:** 10.1186/1749-8090-8-S1-P64

**Published:** 2013-09-11

**Authors:** BM Gersak, JS Sauer

**Affiliations:** 1 LSI SOLUTIONS, 7796 Victor-Mendon Rd., Victor NY 14564, USA

## Background

To test 2-0 B. Braun PremiCron Suture Knot Pull Apart Force of Two Suture Lots fastened with Titanuim fastener Cor Knot® and compare them with USP Class I Min. Avg. 2-0 Suture Knot-Pull Tensile Strength (Doubled for Loop Test) – (USP).

## Methods

Two Suture Lots of 2-0 B. Braun PremiCron Suture: Lot # 1-9364 (24 sutures) and Lot # 110095 (24 sutures) were used and fastened with titanium fastener Cor Knot®. The pull apart force was measured for both of the suture lots.

## Results

The USP Class I Min. Avg. 2-0 Suture Knot-Pull Tensile Strength (Doubled for Loop Test) is defined as 2.88 kgf. The Tensile Strength of Lot # 1-9364 was 4,7761 ± 0,2005 kgf (mean ± std dev) - min 4.2282, max 5.2289, and for Lot # 1-9364 was 5,5151 ± 0,2625 kgf (mean ± std dev) - min 4,0044, max 5,1356, respectively. There were no statistically signiffcant differences between the both lots (F- test, p = 0,102). Comparing tensile strenght for both lots fastened with Cor Knot® with USP tensile strength, knots fatened with Cor Knot® showed higher tensile strengths for all the 48 knots. The minimall tensile strength was in the lot 2-0 PremiCron P/N M0027845, Lot # 110095, and was 4,0044 kgf, which is 1 kgf above the recommended USP strength.

## Conclusion

Titanium fastened 2-0 B. Braun PremiCron Sutures of two tested lots showed much higher Knot Pull Apart Force compared with recommended minimal average USP Class I Min. Avg. 2-0 Suture Knot-Pull Tensile Strength (Doubled for Loop Test). The use of Cor Knot® is safe, fast and stong and may be recommended as a routine knot fastener for general and cardiovascular surgery, especially in combination with endoscopic surgery.

